# Can Tc-99m-PSMA SPECT/CT Be Used as Accessible Alternative for Diagnosis of Biochemically Recurrent Prostate Cancer? A Prospective Study

**DOI:** 10.3390/diagnostics16060895

**Published:** 2026-03-18

**Authors:** Miloš Veljković, Slobodanka Beatović, Tomislav Pejčić, Zoran Bukumirić, Strahinja Odalović, Isidora Grozdić-Milojević, Milica Stojiljković, Jelena Petrović, Ana Ivanovski, Dragana Šobić-Šaranović, Vera Artiko

**Affiliations:** 1 Center for Nuclear Medicine with PET, University Clinical Center of Serbia, 11000 Belgrade, Serbia; beatovic.boba@gmail.com (S.B.); cale_odl@yahoo.com (S.O.); drisidora.grozdic@yahoo.com (I.G.-M.); milica_stoji@yahoo.com (M.S.); jelena_petrovic18@yahoo.com (J.P.); ivanovskia97@gmail.com (A.I.); dsobic2@gmail.com (D.Š.-Š.); vera.artiko@gmail.com (V.A.); 2 Faculty of Medicine, University of Belgrade, 11000 Belgrade, Serbia; tomislav.pejcic@gmail.com (T.P.); zoran.bukumiric@med.bg.ac.rs (Z.B.); 3 Clinic of Urology, University Clinical Center of Serbia, 11000 Belgrade, Serbia; 4 Institute of Medical Statistics and Informatics, 11000 Belgrade, Serbia

**Keywords:** prostate cancer, biochemical recurrence, Tc-99m-PSMA SPECT/CT

## Abstract

**Objective:** To evaluate Tc-99m-PSMA SPECT/CT detection of biochemical recurrence (BCR) of prostate cancer across serum PSA levels in patients treated with radical prostatectomy or radiation therapy, to explore clinical/pathologic predictors of scan positivity and metastatic disease, and to assess its potential role as a pragmatic alternative when PSMA PET/CT is unavailable in resource-limited settings. **Materials and Methods**: In this prospective single-center study, we included 132 men with biochemical recurrence who underwent Tc-99m-PSMA SPECT/CT between January 2024 and December 2025 after predefined inclusion and exclusion criteria were applied, and they were further stratified by primary treatment (radical prostatectomy or radiation therapy). Patients were followed up for up to 6 months after imaging to verify observed findings (histopathology, confirmatory imaging and PSA response) and a logistic regression was applied to identify predictors of scan positivity and metastatic disease. **Results**: In men initially treated with radical prostatectomy, detection increased from 38.9% at PSA 0.2 to <2 ng/mL to 63.2% at 2 to <4 ng/mL, 71.4% at 4 to <7 ng/mL, and 90% at PSA ≥ 7 ng/mL (overall 69.1%). In the radiation therapy cohort, detection was 58.3% at PSA 2 to <4 ng/mL, rising to 85.7% at 4 to <7 ng/mL and 96% at PSA ≥ 7 ng/mL (overall 84.3%). In the multivariable analysis, PSA doubling (log2[PSA]) independently predicted scan positivity and metastatic disease in both cohorts, while seminal vesicle invasion independently predicted metastatic spread in the post-prostatectomy group. **Conclusions:** Tc-99m-PSMA SPECT/CT is a useful tool for detecting prostate cancer BCR, with performance strongly dependent on PSA and higher detection in patients with higher PSA levels. Increasing PSA independently predicted scan positivity and metastatic disease, while seminal vesicle invasion was independently associated with metastatic spread. In settings where PSMA PET/CT is unavailable, Tc-99m-PSMA SPECT/CT may represent a practical alternative, particularly for patients with elevated PSA.

## 1. Introduction

Prostate cancer still imposes a substantial worldwide health burden, being the second most common cancer and the fourth most common cause of death from malignant diseases in males [1]. Recent global analyses confirm the rising incidence of prostate cancer in many regions, as well as projections that the global prostate cancer burden will substantially increase in the coming decades [2,3].

Initial treatment of prostate cancer follows a risk-adapted strategy and is most often managed by either radical prostatectomy or radiation therapy [4,5]. However, despite the advances in treatment, between 20 and 25% of all patients will develop biochemical recurrence (BCR) of prostate cancer within 10 years following initial treatment [6], which is linked to worse disease prognosis and poor survival outcome, especially in patients with PSA doubling time ≤ 1 year, high Gleason score (GS) or high International Society of Urological Pathology (ISUP) grade [7].

BCR of prostate cancer is defined as the elevation of serum prostate-specific antigen (PSA) to levels ≥ 0.2 ng/mL after radical prostatectomy, confirmed by another measurement of elevated PSA after a few weeks, or ≥2 ng/mL above post-treatment PSA nadir after radiation therapy [8]. Early detection of BCR in patients with prostate cancer plays a pivotal role in the management of disease outcomes. Traditionally, contrast-enhanced magnetic resonance imaging (MRI) and computed tomography (CT) are most commonly used; however, their diagnostic yield for detection of BCR is rather low, especially in patients with low-serum PSA [9,10].

Over the past decade, the use of prostate-specific membrane antigen (PSMA)-labeled molecular imaging has advanced rapidly, becoming an increasingly important tool in the diagnosis of prostate cancer [11,12]. PSMA is a type II transmembrane glycoprotein with a large extracellular domain that is located on the surface of normal prostate cells, but is up-regulated 10-fold or more times in prostate cancer [13], which makes it an attractive target for targeted molecular imaging or therapy. As such, multiple PSMA inhibitors or small PSMA-ligands have been developed in recent years, usually labeled with positron emitters such as Ga-68 or F-18 for diagnosis of prostate cancer using positron emission tomography with computed tomography (PET/CT), or labeled with Lu-177 or Ac-225 for targeted radionuclide therapy of prostate cancer [14,15,16]. Routine clinical use of these radiopharmaceuticals in diagnosis and therapy have significantly contributed to prostate cancer management, although their availability still varies between regions and institutions.

While performance of PSMA PET/CT in diagnosis of prostate cancer has been extensively studied and guidelines for its use in clinical practice have been published [17], there is a rather limited amount of studies that have explored the performance of single-photon emission computed tomography (SPECT/CT) using Tc-99m-PSMA, especially prospective studies stratified by serum PSA levels.

SPECT/CT systems are more commonly available and already installed in many nuclear medicine departments, whereas PET/CT capacity is often more centralized [18]. This is especially relevant for resource-limited settings where clinicians can leverage the established global infrastructure of gamma cameras and Tc-99m generators, which are widely used for routine nuclear medicine procedures [19]. Tc-99m can be produced in large amounts using relatively inexpensive Mo-99/Tc-99m generators, which provide enough activity for routine, high-volume clinical imaging. Its favorable emission properties and longer half-life (6 h) facilitate wider distribution, making Tc-99m-SPECT/CT a low-cost, globally adopted diagnostic tool [19,20]. In addition, the availability of kit-based Tc-99m-PSMA radiopharmaceuticals further lowers the operational barriers, while increasing the usefulness of Tc-99m SPECT/CT for prostate cancer imaging [21]. In contrast, PSMA PET/CT requires either cyclotron-produced tracers or costly Ge-68/Ga-68 generators that yield a limited number of patient doses per elution, and the short half-life of Ga-68 limits large-scale distribution and routine implementation [19].

Given the continuous rise in prostate cancer incidence globally as well as the need for early detection of BCR, the objective of our study was to evaluate diagnostic performance of Tc-99m-PSMA SPECT/CT in the diagnosis of BCR of prostate cancer at different serum PSA levels, evaluate important predictors of Tc-99m-PSMA SPECT/CT and determine whether this diagnostic method could serve as an affordable and widely available alternative to PSMA PET/CT, particularly in developing countries where advanced imaging is often limited.

## 2. Materials and Methods

### 2.1. Study Design and Population

**Figure 1:**
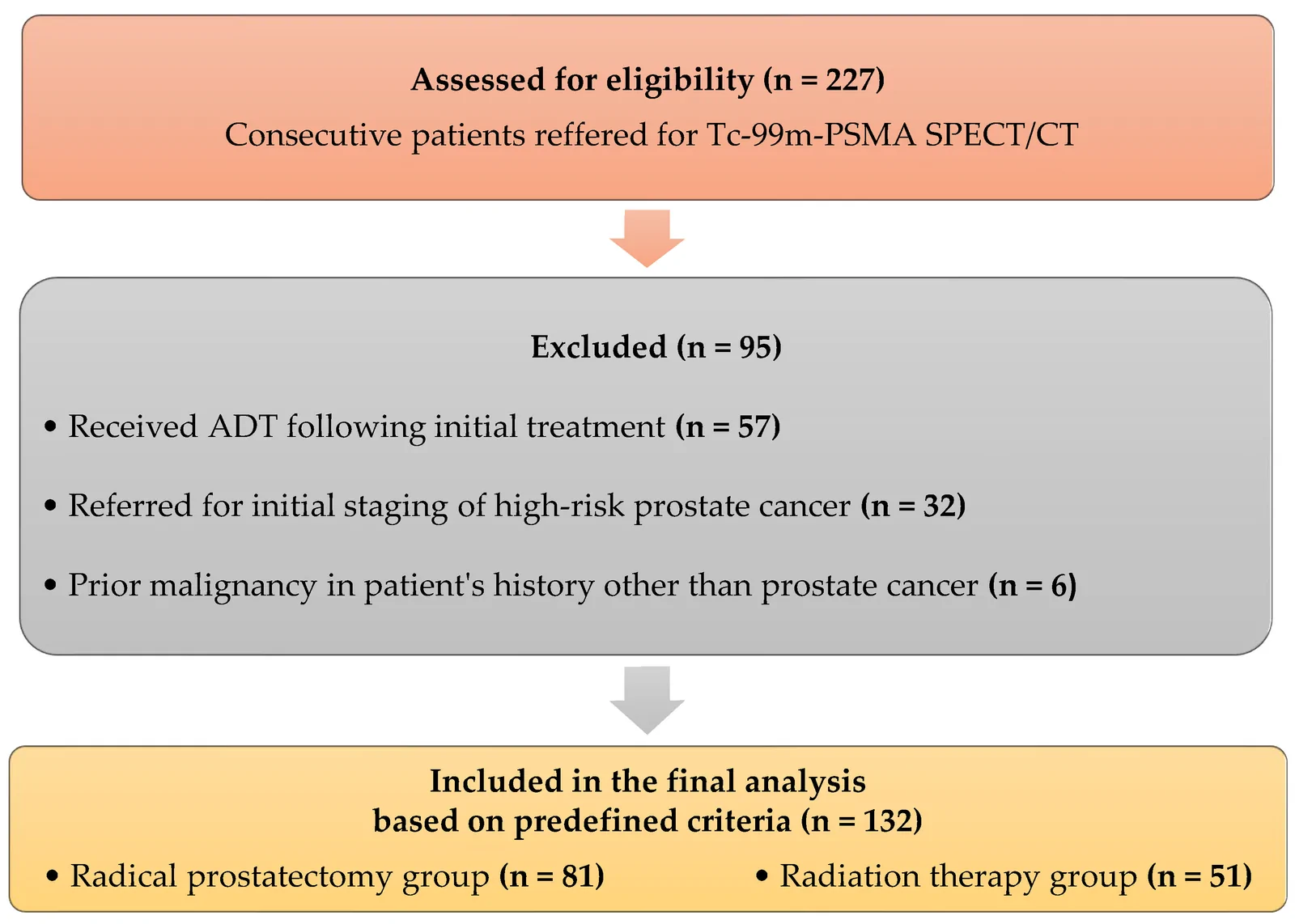
Flow diagram of patient selection.

In this prospective single-center study, 227 consecutive patients with prostate cancer were referred to our center between January 2024 and December 2025 for further evaluation with Tc-99m-PSMA SPECT/CT. After application of the predefined inclusion and exclusion criteria, 95 patients were excluded from the study, while 132 men with biochemical recurrence (BCR) of prostate cancer were included in the final analysis (median PSA 5.95 ng/mL, IQR 3.22–11.2 ng/mL), as shown in Figure 1.

The inclusion criteria of the study were:

1. Histopathologically confirmed prostate cancer and biochemical recurrence of disease ≥ 6 months after primary treatment, defined as: (1) after radical prostatectomy—PSA ≥ 0.2 ng/mL, confirmed by a second measurement ≥4 weeks apart, or (2) after radiation therapy—PSA ≥ 2.0 ng/mL above the post-treatment PSA nadir;
2. Absence of other malignancies in the patient’s history;
3. Availability of follow-up data for up to 6 months after Tc-99m-PSMA SPECT/CT, or sufficient enough to verify Tc-99m-PSMA SPECT/CT findings (e.g., histopathology, follow-up imaging and PSA changes).

The exclusion criteria of the study were:

1. Patients who received additional therapy after primary treatment (androgen-deprivation therapy (ADT) or chemotherapy);
2. Severe renal failure in the patient’s history (eGFR of 15 to 29 mL/min/1.73 m^2^ or lower) [22].

Patients included in the study were stratified into two groups, based on initial treatment of prostate cancer (radical prostatectomy and radiation therapy). This study was approved by the Institutional Ethics Committee of the University Clinical Center of Serbia (initial approval No. 668/6, 19 April 2018; continuation approval No. 1500/45, 29 September 2025). Written informed consent was obtained from all patients. Patient characteristics are shown in Table 1.

### 2.2. Radiopharmaceutical

Tc-99m-PSMA was prepared using PARS-TECTO PSMA cold kit (Pars Isotope Co., Tehran, Iran) according to manufacturer’s instructions. Each vial contains 20 µg Tc-99m-HYNIC-PSMA. Quality control procedures, as well as a radiochemical purity assessment, were performed according to institutional protocols to ensure reliable tracer performance in all patients that were imaged. The final product was administered intravenously within 30 min of its preparation.

### 2.3. Tc-99m-PSMA SPECT/CT

Tc-99m-PSMA SPECT/CT was performed in all 132 men who were included in our study, 3–4 h after IV administration of 7.4 MBq/kg (0.2 mCi) of Tc-99m-PSMA per kilogram of body weight. Prior to the image acquisition, patients were instructed to void. Image acquisition was performed using a hybrid gamma camera Simbya Intevo (Siemens, Erlangen, Germany) using low energy high-resolution collimator with a photopeak of Tc-99m−140 keV and matrix size of 128 × 128. Acquisition protocol: (1) Planar whole body scintigrams in AP and PA projections; (2) whole body SPECT (from skull base to mid-thigh) in 360°, “step and shoot” mode, 30 s per image; (3) low-dose CT (120 keV, 40 mAs, slice thickness 3 mm, pitch 1.5, rotation time 0.5 s) in the region of SPECT. If whole body planar scintigrams in AP and PA projections revealed sites of increased Tc-99m-PSMA uptake in the regions not scanned normally by the default SPECT/CT protocol (skull base to mid-thigh), the scanned region was extended to include revealed sites of increased Tc-99m-PSMA uptake. This extended field-of-view approach ensured that atypical metastatic sites were not missed, providing comprehensive coverage beyond the standard acquisition range when necessary.

### 2.4. Image Interpretation

Interpretation of acquired images was performed independently by two experienced nuclear medicine physicians. If there was discordance in the findings between the two physicians, a third nuclear medicine physician was consulted and a final interpretation was reached as a consensus. To minimize interpretation bias, all physicians were blinded to patients’ serum PSA levels during image review.

As there is no validated dedicated scoring system for Tc-99m-PSMA SPECT/CT, image interpretation was based on a visual and morphologic assessment using combined SPECT and low-dose CT findings. Image interpretation was performed on a lesion-by-lesion basis and then summarized at the patient level. Interpretation included assessment of lesion intensity, focality, anatomic location, and correlation with low-dose CT morphology. Before applying lesion-specific interpretation criteria, all areas of tracer uptake were assessed on fused SPECT/CT to exclude physiological tracer distribution and benign or nonspecific causes of uptake. Lymph nodes in the pelvis and retroperitoneum were considered positive if they demonstrated focal tracer uptake higher than blood-pool activity, whereas lymph nodes in other nodal stations (e.g., mediastinal or cervical) were considered positive when focal tracer uptake was visually higher than liver activity. Prostate bed lesions after radical prostatectomy were considered positive if they showed focal tracer uptake higher than blood-pool activity that could not be attributed to urinary/bladder activity. Prostatic lesions after radiation therapy were considered positive if tracer uptake was visually equal to or higher than liver activity. Bone and soft-tissue lesions were interpreted in conjunction with low-dose CT findings; if a corresponding lesion was identified on CT (e.g., sclerotic bone lesion or soft-tissue nodule), it was considered positive when PSMA uptake was higher than blood-pool activity. In the absence of a clear CT correlate, lesions were considered positive when tracer uptake was visually equal to or higher than liver activity.

For lesion distribution, images were categorized as local recurrence (recurrence in prostate/prostate bed, and/or pelvic lymph node) and metastatic recurrence (recurrence in extra-pelvic lymph nodes, bones and/or visceral organs).

### 2.5. Follow-Up

Patients were followed up clinically for up to 6 months after Tc-99m-PSMA SPECT/CT or until sufficient data was collected to verify Tc-99m-PSMA SPECT/CT findings. Results of the Tc-99m-PSMA SPECT/CT scan were considered true positive if a histopathological report or follow-up imaging method (CT, MRI or a follow-up Tc-99m-PSMA SPECT/CT scan) confirmed previously reported findings. PSA response to therapy after Tc-99m-PSMA SPECT/CT was not used as the sole criterion to establish lesion positivity, but was rather considered supportive only when accompanied by follow-up imaging. Results were considered false positive if a histopathological finding was not consistent with prostate cancer recurrence or metastases, or when follow-up imaging failed to confirm the reported lesions. Because all patients met the BCR criteria for inclusion of the study, scans were classified as false negatives if there was no pathological uptake on Tc-99m-PSMA SPECT/CT; likewise, because all patients met BCR criteria, true negative cases could not be confidently defined using the available reference standard. This multi-level validation strategy was implemented to ensure accurate classification of imaging outcomes and reduce the inherent uncertainty associated with limited histopathological sampling in metastatic prostate cancer.

### 2.6. Statistical Analysis

Statistical analyses were performed with IBM SPSS Statistics for Windows, Version 25.0. (IBM Corp, Armonk, NY, USA) and with the R software package (R Core Team, 2021, version 4.3.1). To identify variables associated with Tc-99m-PSMA SPECT/CT positivity, univariate and multivariate logistic regression analyses were performed separately for each treatment group. Serum PSA was log2-transformed so that the odds ratio (OR) represents the change in odds associated with doubling of PSA. In the radical prostatectomy group, the variables used were PSA, ISUP grade and pathology features (perineural invasion, lymphovascular invasion, surgical margin status, extracapsular extension and seminal vesicle invasion). In the radiation therapy group, PSA and ISUP grade were included in the analysis. In addition, a logistic regression was used to evaluate predictors of disease distribution on Tc-99m-PSMA SPECT/CT within each treatment group, using the same covariates available for each group. Results are reported as ORs with 95% confidence intervals (CI). A calculated *p* value < 0.05 was considered statistically significant.

## 3. Results

### 3.1. Radical Prostatectomy Group

The radical prostatectomy cohort included 81 patients with a median age of 71.0 (68.0–74.0) years and median PSA of 4.5 (IQR 2.2–10.8) ng/mL at the time of Tc-99m-PSMA SPECT/CT imaging. Most patients had intermediate- to high-grade disease, with ISUP grade 3 being the most common (54.3%), followed by ISUP grade 2 (22.2%) and ISUP grade 5 (14.6%). Unfavorable pathology features were frequent in our cohort, including extracapsular extension in 24.7% of the cases and seminal vesicle invasion in 33.3% of the cases. The pathology features reported in Table 1 are not mutually exclusive, as 64.2% of patients had ≥2 features, while 29.6% of patients had ≥3 features, with perineural and lymphovascular invasion being the most common.

#### 3.1.1. Radical Prostatectomy Group—Tc-99m-PSMA SPECT/CT Detection Rates of BCR at Different PSA Levels

Tc-99m-PSMA SPECT/CT detection rates of BCR in the radical prostatectomy group at different PSA levels are shown in Table 2.

Among the 81 patients included in the radical prostatectomy group, 18 men had PSA 0.2 to <2 ng/mL (median 1.00 [IQR 0.74–1.38]), and Tc-99m-PSMA SPECT/CT was positive in 7/18 patients (38.9%); 19 men were included in PSA 2 to <4 ng/mL group (median 3.25 [IQR 2.70–3.55]) with positive scans in 12/19 (63.2%), while 14 men were in PSA 4 to <7 ng/mL group (median 5.53 [IQR 4.68–6.54]), and Tc-99m-PSMA SPECT/CT was positive in 10/14 (71.4%). Finally, 30 men had PSA ≥ 7.0 ng/mL (median 13.89 [IQR 9.13–22.35]), with positive Tc-99m-PSMA SPECT/CT in 27/30 (90%) patients. Overall, the detection rate in this subgroup was 56/81 (69.1%), showing a progressive increase in Tc-99m-PSMA SPECT/CT positivity with rising PSA.

#### 3.1.2. Radical Prostatectomy Group—Most Common Recurrence Sites of Prostate Cancer and Disease Burden

**Figure 2:**
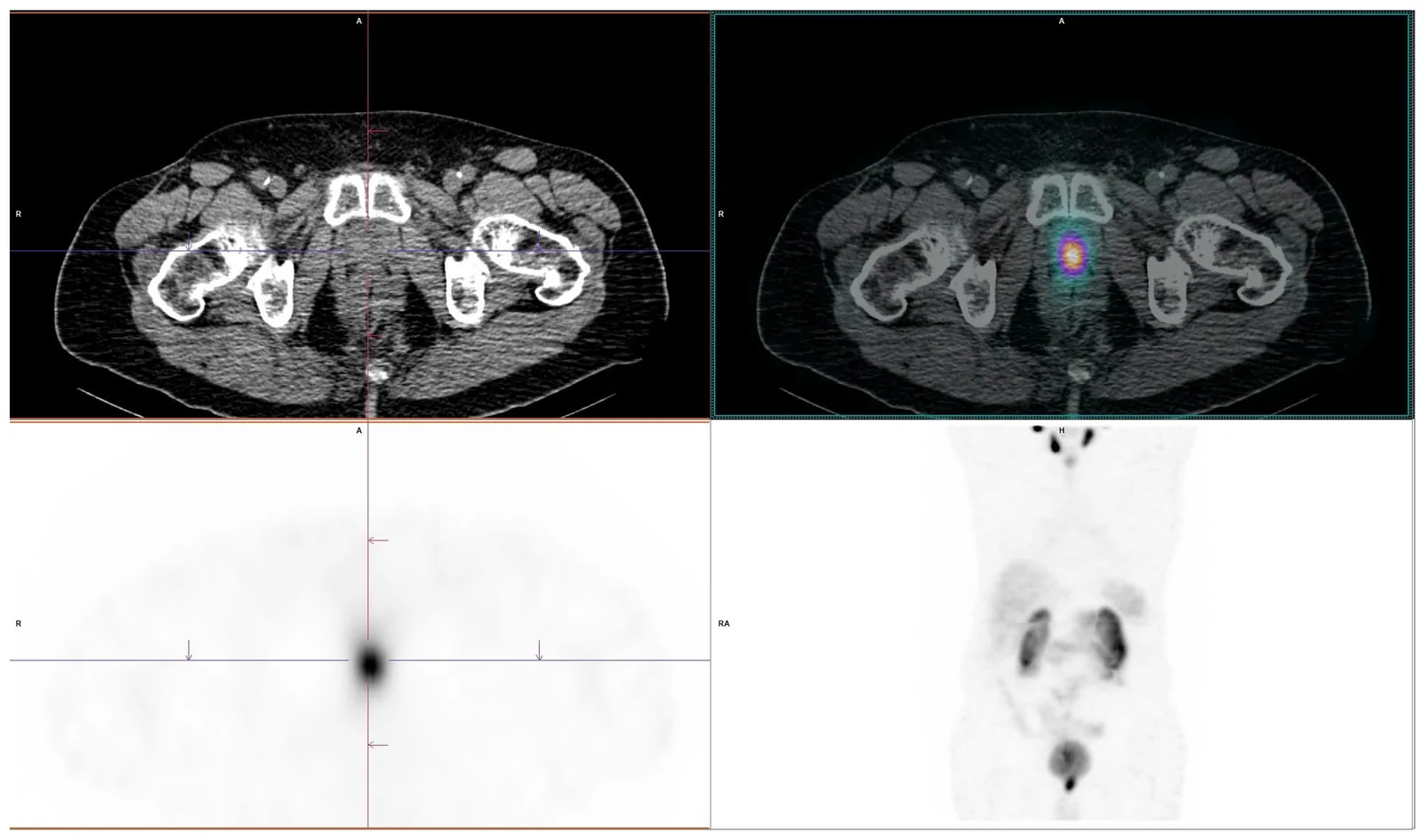
A 75-year-old man with BCR of prostate cancer after radical prostatectomy (ISUP grade 2) underwent Tc-99m-PSMA SPECT/CT at a PSA of 2.95 ng/mL, which demonstrated pathological tracer uptake in the prostate bed, highly suspicious for local recurrence. Multiparametric MRI was performed 2 weeks later, which revealed a focal area of increased T2 signal with restricted diffusion in the prostate bed, also suspicious for recurrent disease. Based on the concordant SPECT/CT and MRI findings, the patient received salvage radiotherapy to the prostate bed, resulting in a subsequent decline in serum PSA.

Among the 56 positive Tc-99m-PSMA SPECT/CT patients, the most common recurrence sites were pelvic lymph nodes in 39 patients (69.6%), followed by bones in 20 patients (35.7%), extra-pelvic lymph nodes in 13 patients (23.2%), prostate bed in 12 patients (21.4%) and visceral organs in three patients (5.4%). These sites were not mutually exclusive as 24/56 PSMA-positive patients (42.9%) demonstrated involvement of two or more sites; seven patients (29.2%) had both pelvic lymph node and bone involvement, while five patients (20.8%) had involvement of both prostate bed and pelvic lymph nodes. Based on the detected disease burden, local disease (prostate bed and/or pelvic lymph node involvement) was observed in 25/56 patients (44.6%), while metastatic disease (extra-pelvic lymph nodes, bones, visceral organs) was present in 31/56 patients (55.4%). One of our patients from radical prostatectomy group is shown in Figure 2.

#### 3.1.3. Radical Prostatectomy Group—Predictors of Tc-99m-PSMA SPECT/CT Positivity and Disease Burden (Logistic Regression)

Univariate and multivariate logistic regression analyses were performed to determine predictors of Tc-99m-PSMA SPECT/CT positivity and metastatic disease spread in radical prostatectomy group, as shown in Table 3.

PSA was log2-transformed so that the odds ratio (OR) represents the change in odds associated with doubling of PSA. In the univariate logistic regression, each PSA doubling was associated with 68% higher odds of Tc-99m-PSMA SPECT/CT positivity (OR 1.68 per PSA doubling, 95% CI 1.19–2.35; *p* = 0.003). ISUP grade per 1-grade increase showed a trend towards Tc-99m-PSMA SPECT/CT positivity (OR 1.67 per 1-grade increase, 95% CI 0.98–2.82; *p* = 0.058). All of the pathological features (PNI, LVI, PSM, ECE and SVI) were not significantly associated with Tc-99m-PSMA SPECT/CT positivity (all *p* > 0.05).

Predictors that were statistically significant or showed a trend towards Tc-99m-PSMA SPECT/CT positivity were included in multivariate analysis. In multivariate regression analysis including log2(PSA) and ISUP grade (per 1-grade increase), PSA doubling remained independently associated with scan positivity (aOR 1.62, 95% CI 1.14–2.30; *p* = 0.007), whereas ISUP grade was not statistically significant after adjustment (aOR 1.55, 95% CI 0.90–2.65; *p* = 0.11).

The univariate logistic regression was also performed to evaluate predictors of metastatic disease on Tc-99m-PSMA SPECT/CT. PSA doubling was associated with 74% higher odds of metastatic disease spread (OR 1.74 per PSA doubling, 95% CI 1.19–2.56; *p* = 0.004). Seminal vesicle invasion (SVI) was also significantly associated with metastatic spread (OR 4.70, 95% CI 1.60–13.80; *p* = 0.005), whereas ISUP grade per 1-grade increase showed a trend towards metastatic spread of the disease in the univariable analysis (OR 1.55 per 1-grade increase, 95% CI 0.93–2.59; *p* = 0.09). Other pathological features were not significantly associated with metastatic spread (all *p* > 0.05).

In the multivariable model including log2(PSA), ISUP grade, and SVI, both PSA (aOR 1.54 per PSA doubling, 95% CI 1.02–2.33; *p* = 0.04) and SVI (aOR 3.70, 95% CI 1.19–11.50; *p* = 0.02) remained independent predictors of metastatic disease spread, but ISUP grade per 1-grade increase was not statistically significant (aOR 1.15, 95% CI 0.66–2.01; *p* = 0.62).

### 3.2. Radiation Therapy Group

The radiation therapy cohort included 51 patients with a median age of 72 (IQR 69.5–77.0) years and a median PSA of 6.6 (IQR 4.1–11.8) ng/mL at the time of Tc-99m-PSMA SPECT/CT imaging. Over one-third of patients in this cohort had ISUP grade 3 disease (35.3%), followed by ISUP grade 2 (25.5%) and ISUP grade 4 (17.6%).

#### 3.2.1. Radiation Therapy Group—Tc-99m-PSMA SPECT/CT Detection Rates of BCR at Different PSA Levels

Tc-99m-PSMA SPECT/CT detection rates of BCR in the radiation therapy group at different PSA levels are shown in Table 4.

In the radiation therapy group, a total of 51 patients were included in the study. Twelve patients had PSA 2 to <4 ng/mL (median 2.79 [IQR 2.44–3.42]), and Tc-99m-PSMA SPECT/CT was positive in 7/12 patients (58.3%), 14 men had PSA 4 to <7 ng/mL (median 5.63 [IQR 4.93–6.38]) with positive Tc-99m-PSMA SPECT/CT in 12/14 (85.7%), while 25 men had PSA ≥ 7.0 ng/mL (median 11.88 [IQR 8.09–21.40]) with positive Tc-99m-PSMA SPECT/CT in 24/25 (96%) patients. Overall, the detection rate in the radiation therapy group was 43/51 (84.3%), demonstrating an increasing Tc-99m-PSMA SPECT/CT positivity with rising PSA and an overall higher detection rate than in the radical prostatectomy group (69.1%).

#### 3.2.2. Radiation Therapy Group—Most Common Recurrence Sites of Prostate Cancer and Disease Burden

**Figure 3:**
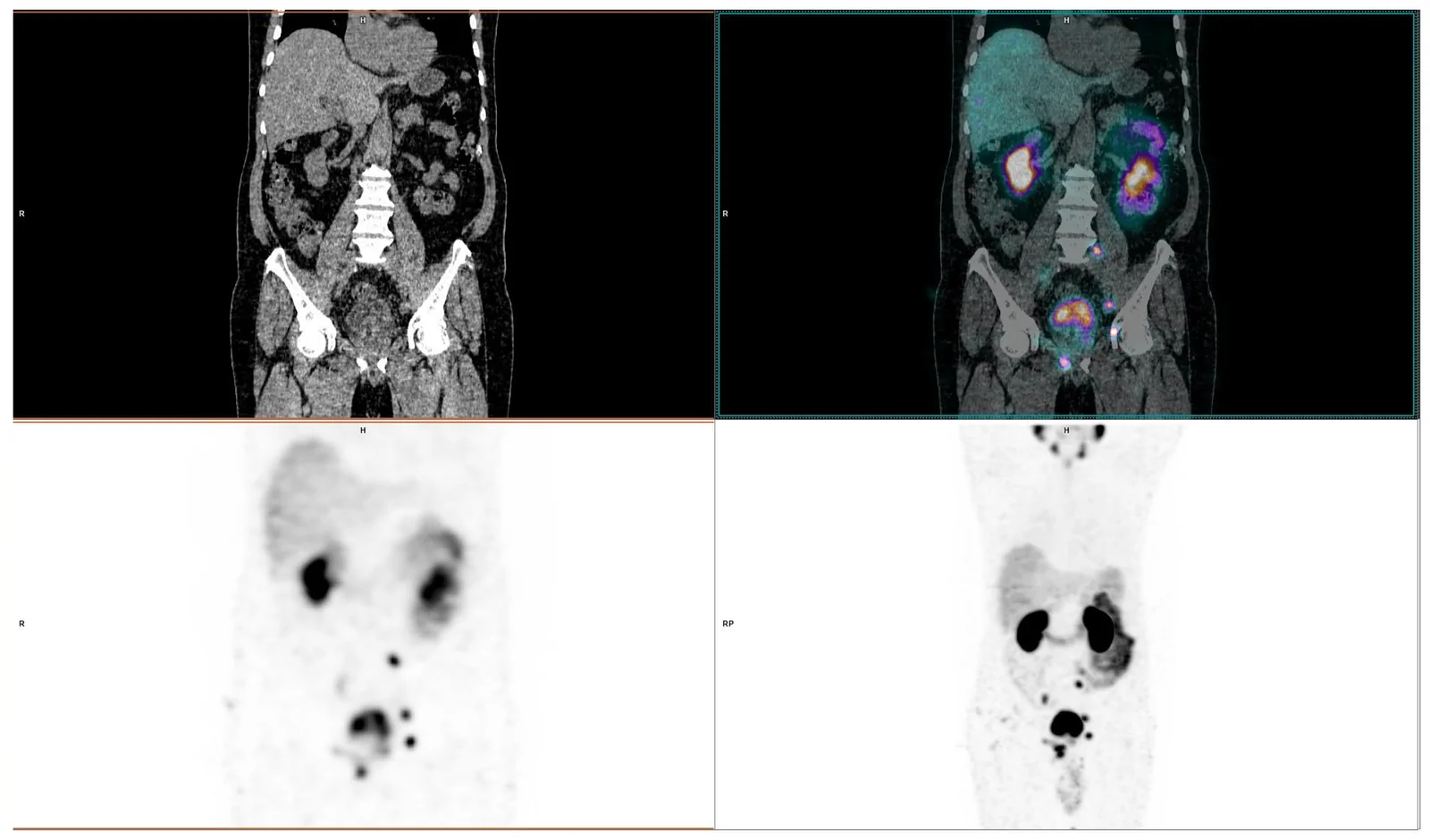
A 61-year-old man with BCR of prostate cancer after radiation therapy (ISUP grade 3) underwent Tc-99m-PSMA SPECT/CT at a PSA of 6.33 ng/mL, which revealed multiple sites pathological tracer uptake in the pelvic and retroperitoneal lymph nodes, as well as pelvic bones, compatible with prostate cancer metastases. ADT was initiated afterwards as a part of routine clinical management, which resulted in a decrease in serum PSA. On follow-up abdominopelvic MDCT after 5 months, reported enlarged lymph nodes on Tc-99m-PSMA SPECT/CT were no longer visible, indicating disease regression; pelvic bones still had previously reported sclerotic lesions, stable in size.

Tc-99m-PSMA SPECT/CT revealed sites of recurrence in 43 patients, and the most common recurrence sites were prostate glands in 29 patients (67.4%), followed by pelvic lymph nodes and bones in 11 patients each (25.6%), distant lymph nodes in six patients (14%), and visceral organs in four patients (9.3%). Sites of recurrence were also not mutually exclusive, as five patients (11.6%) presented with disease recurrence in prostate and pelvic lymph nodes, while two (4.7%) patients had disease recurrence in both prostate and bones. Based on the detected disease extent, local disease (prostate gland recurrence and/or pelvic lymph node involvement) was observed in 27/43 patients (62.8%), while metastatic disease (extra-pelvic lymph nodes, bones, visceral organs) was detected in 16/43 patients (37.2%). One of our patients from radiation therapy group is shown in Figure 3.

#### 3.2.3. Radiation Therapy Group—Predictors of Tc-99m-PSMA SPECT/CT Positivity and Disease Burden (Logistic Regression)

Univariate and multivariate logistic regression analyses were performed to determine if PSA (as log2(PSA)—per doubling) and ISUP grade per 1-grade increase are significant predictors of Tc-99m-PSMA SPECT/CT positivity and metastatic disease spread in the radiation therapy group, as shown in Table 5.

In the univariate logistic regression, each PSA doubling was significantly associated with Tc-99m-PSMA SPECT/CT positivity (OR 2.87 per PSA doubling, 95% CI 1.07–7.71; *p* = 0.036) and with metastatic disease spread (OR 2.32 per PSA doubling, 95% CI 1.10–4.86; *p* = 0.026), while ISUP grade per 1-grade increase did not reach statistical significance for Tc-99m-PSMA SPECT/CT positivity (*p* = 0.32) or metastatic disease spread (*p* = 0.77).

In multivariate regression analysis including log2(PSA) and ISUP grade (per 1-grade increase), PSA doubling remained independently associated with scan positivity (aOR 3.07, 95% CI 1.12–8.41; *p* = 0.029) and metastatic disease spread (aOR 2.35, 95% CI 1.11–4.97; *p* = 0.025), whereas ISUP grade remained statistically insignificant.

## 4. Discussion

In our prospective study, we evaluated Tc-99m-PSMA SPECT/CT detection rates of BCR at different PSA levels in patients initially treated either with radical prostatectomy or radiation therapy. In the radical prostatectomy group, the detection rates were 38.9%, 63.2%, 71.4% and 90% for PSA strata 0.2 to <2, 2 to <4, 4 to <7 and ≥7 ng/mL respectively, with an overall detection rate of 69.1%. In the radiation therapy group, the detection rates were 58.3%, 85.7% and 96% for patient groups with serum PSA 2 to <4, 4 to <7 and ≥7 ng/mL respectively, with an overall detection rate of 84.3%. The overall detection rate of BCR in both the radical prostatectomy and radiation therapy groups was 75% (99/132).

There is a limited number of previously published studies that have evaluated the performance of Tc-99m-PSMA SPECT/CT for BCR detection at different serum PSA thresholds, making direct comparisons challenging. However, most of these retrospective studies reported overall findings that were broadly comparable to those of our prospective study [23,24,25,26]. The reported overall detection rates of BCR in these retrospective cohorts ranged between 70.7% and 78%, which is highly comparable to the 75% overall detection rate observed in our study. Despite these similarities, there were differences in detection rates across varying PSA strata, particularly in the setting of PSA < 2 ng/mL. The most likely explanation is that most of the aforementioned retrospective studies [23,24,25] also included patients who had received ADT in their cohorts which can significantly alter PSMA expression and confound the relationship between PSA level and scan positivity [27,28]. In a retrospective study by Werner et al. [29], which included 152 patients with BCR after either prostatectomy or radiation therapy (with no subgroup analysis based on initial treatment), the observed overall detection rate of Tc-99m-PSMA SPECT/CT was 57.6%, which is lower than the combined detection rate observed in our study (75%). This can be explained by the fact that the detection rate of Tc-99m-PSMA SPECT/CT is significantly influenced by serum PSA levels, as they had a higher number of patients with lower serum PSA levels (99 patients with PSA < 4 ng/mL), while in our study there were 49 patients with serum PSA > 4 ng/mL (37 patients with serum PSA between 0.2 to <4 ng/mL in the radical prostatectomy group and 12 patients with serum PSA 2 < 4 ng/mL in the radiation therapy group).

Our results demonstrate that log2(PSA)—each twofold (doubling) increase in PSA at the time of imaging—is an independent predictor of Tc-99m-PSMA SPECT/CT positivity in both groups (radical prostatectomy: aOR 1.62, *p* = 0.007; radiation therapy: aOR 3.07, *p* = 0.029) and also of metastatic disease (radical prostatectomy: aOR 1.54, *p* = 0.04; radiation therapy: aOR 2.35, *p* = 0.025), consistent with results from previously published studies [22,25]. In the radical prostatectomy group, SVI was the strongest independent pathological predictor of metastatic disease on Tc-99m-PSMA SPECT/CT (aOR 3.70, *p* = 0.02). Luining W et al. [30] reported similar findings in a large retrospective cohort of 2193 patients with prostate cancer, where SVI was significant predictor of metastatic disease (OR 4.36, *p* < 0.001), while ECE was not (OR 1.55, *p* = 0.12). Potter S et al. [31] also identified SVI as an adverse pathological factor for recurrence and noted that, once BCR develops, men with SVI more often present with distant rather than local metastases. Secin et al. [32] evaluated 4441 patients after radical prostatectomy and found that patients with SVI had a 10-year BCR-free probability of 36%, while those with SVI plus nodal metastases only had a 10% BCR-free probability after 10 years, suggesting a more aggressive phenotype of disease recurrence.

An important consideration in interpreting our results is that the radical prostatectomy and radiation therapy groups are not directly comparable, as they represent distinct clinical populations following different primary treatment modalities. Although the radiation therapy group had a higher median PSA level at the time of imaging (6.6 vs. 4.5 ng/mL), the radical prostatectomy group demonstrated several features suggestive of a more aggressive disease biology. Specifically, ISUP grade 3–5 tumors were more frequently observed in the radical prostatectomy cohort, and a substantial proportion of these patients presented with adverse pathological features, including positive surgical margins (38.3%), extracapsular extension (24.7%), and seminal vesicle invasion (33.3%). Moreover, although BCR was more frequently identified in the radiation therapy group (84.3% vs. 69.1%), the radical prostatectomy group had a higher proportion of patients with metastatic disease (55.4% vs. 37.2%), further supporting the presence of a biologically unfavorable subgroup within this population. Therefore, the observed differences between the two cohorts should be interpreted with caution and should not be regarded as a direct head-to-head comparison of imaging performance between the two treatment modalities.

The performance of PSMA PET/CT, using both Ga-68 and F-18 radiopharmaceuticals, has been extensively researched and currently represents the standard of care for the detection of BCR. A large systematic review by Mazzone et al. [33] that included 8119 patients reported sample-weighted mean detection rates of BCR in patients with PSA < 1 ng/mL of 53% for Ga-68-PSMA-11, 42% for F-18-DCFPyl and 66% for F-18-Flotufolastat, which are superior to the detection rates observed in our study with Tc-99m-PSMA SPECT/CT (a 38.9% detection rate at PSA 0.2 to <2 ng/mL in the radical prostatectomy group). Afshar-Oromieh et al. [34] conducted a large multi-center evaluation of Ga-68-PSMA PET/CT in the detection of recurrent prostate cancer at varying serum PSA levels and reported probability of pathological scan of 58% and 72% for serum PSA 0.2–0.5 ng/mL and 0.5–1 ng/mL respectively, further rising up to 93% in patients with serum PSA > 10 ng/mL. In comparison to our reported findings, it is evident that at low-serum PSA levels PSMA PET/CT demonstrates superior detection performance than Tc-99m-PSMA SPECT/CT due to higher spatial resolution of PET/CT compared to SPECT/CT. However, as serum PSA levels rise, Tc-99m-PSMA SPECT/CT also achieves high-detection efficiency as observed in our study, reaching 90% and 96% in radical prostatectomy and radiation therapy groups respectively, in patients with PSA ≥ 7 ng/mL.

A very limited number of studies report head-to-head comparison of Tc-99m-PSMA SPECT/CT and PSMA PET/CT in the detection of prostate cancer. In one such study reporting on detection rates in 28 patients, 25/28 (89.2%) patients had abnormal Ga-68-PSMA PET/CT findings, while 20/28 (71.4%) patients had pathological Tc-99m-PSMA SPECT/CT findings [35]. In the same study, it was also reported that at serum PSA > 2.1 ng/mL no patient had discordant results between two imaging modalities, even though Ga-68-PSMA PET/CT detected more lesions [35]. In a study by Lawal et al. [36] the total number of patients was only 14 and it was reported that Ga-68-PSMA PET/CT detected more lesions than Tc-99m-PSMA SPECT/CT, with lymph node size being a significant predictor of lesion detection—all lymph nodes with size > 10 mm were detected by both modalities, while only 28% of nodes < 10 mm were detected by Tc-99m-PSMA SPECT/CT. Similar results were reported in a study by Singh et al. [37], in which Ga-68-PSMA PET/CT detected lesions in 10/10 patients included in the study, while Tc-99m-PSMA SPECT/CT detected lesions in 9/10 patients, with Ga-68-PSMA PET/CT detecting more lesions. All three aforementioned studies conclude that Tc-99m-PSMA SPECT/CT can be a potential alternative to PSMA PET/CT for prostate cancer detection, while illustrating the expected diagnostic performance gap between PSMA PET and PSMA SPECT.

While the cost of imaging patients with Ga-68 or F-18 PSMA PET/CT and Tc-99m-PSMA SPECT/CT varies between different countries and medical centers, with the strict pricing not publicly available, it is general knowledge that Tc-99m-PSMA SPECT/CT is a more widely available and affordable tool for prostate cancer diagnosis when compared to PSMA PET/CT [19,20,21,38,39]. Given the global rise in prostate cancer incidence, cost-effective diagnostic tools like Tc-99m-PSMA SPECT/CT can play a crucial role in expanding early detection of prostate cancer and its recurrence [40,41].

Our study has certain limitations. This was a single-center study, which limited the number of men with prostate cancer that underwent Tc-99m-PSMA SPECT/CT for detection of BCR, which hinders the ability to perform direct head-to-head comparisons between two treatment modalities and their subgroups. Another limitation is the lack of histopathological confirmation of each lesion detected on Tc-99m-PSMA SPECT/CT. This is difficult to achieve in routine clinical practice, especially in patients with multiple metastases, so we had to use PSA response to therapy (which can lead to a possible verification bias) and follow-up imaging in addition to histopathological results of lesions detected on Tc-99m-PSMA SPECT/CT. From a technological standpoint, a key limitation of SPECT/CT is the lack of a broadly accepted and harmonized semiquantitative metric (such as SUVmax and SUVmean) in contrast to PET/CT [39]. Historically, SPECT/CT has been viewed as less suitable for quantitative imaging, although recent advances in detector technology and reconstruction algorithms have made PET-like quantification technically feasible [40]. Nevertheless, quantitative SPECT/CT workflows remain inconsistently implemented across institutions, and standardized quantification is not yet routine [39]. As a result, even when modern systems can generate SUV-like measurements, uptake assessment is often less reproducible, limiting objective evaluation of tracer avidity and reducing the reliability of response monitoring—areas where PET/CT continues to offer a clear advantage [39].

## 5. Conclusions

Tc-99m-PSMA SPECT/CT is a useful tool for the detection of BCR, with observed overall detection rates of 69.1% after radical prostatectomy and 84.3% after radiation therapy. The detection rate is strongly PSA-dependent, ranging from 38.9% at PSA 0.2 to <2 ng/mL to >90% at PSA ≥ 7 ng/mL. Rising serum PSA independently predicted Tc-99m-PSMA SPECT/CT positivity and metastatic disease, while seminal vesicle invasion was independently associated with metastatic spread. In resource-limited settings where PSMA PET/CT is unavailable, Tc-99m-PSMA SPECT/CT may serve as a practical alternative, especially for patients with higher PSA. Further multicenter studies on a larger number of patients are required to establish the role of Tc-99m-PSMA SPECT/CT in the diagnostic algorithm of prostate cancer and its recurrence.

## Figures and Tables

**Table 1:** Patient characteristics.

**Characteristics**		
Patients (*n*)	132	
**Initial treatment**	**Radical prostatectomy**	**Radiation therapy**
Patients, n (%)	81 (61.4%)	51 (38.6%)
Age (years), median (IQR)	71.0 (68.0–74.0)	72.0 (69.5–77.0)
PSA (ng/mL), median (IQR)	4.5 (2.2–10.8)	6.6 (4.1–11.8)
**ISUP grade**	***n (%)***	***n (%)***
Grade 1	4 (4.9%)	6 (11.8%)
Grade 2	18 (22.2%)	13 (25.5%)
Grade 3	44 (54.3%)	18 (35.3%)
Grade 4	3 (3.7%)	9 (17.6%)
Grade 5	12 (14.6%)	5 (9.8%)
**Pathology features**	***n (%)***	
Perineural invasion	49 (60.5%)	n/a
Lymphovascular invasion	34 (42%)	n/a
Positive surgical margin	31 (38.3%)	n/a
Extra-capsular extension	20 (24.7%)	n/a
Seminal vesicle invasion	27 (33.3%)	n/a

**Table 2:** Radical prostatectomy group—Tc-99m-PSMA SPECT/CT detection rates of BCR in different PSA groups.

PSA Group (ng/mL)	Patients (n)	PSA Median (IQR)	Positive Tc-99m-PSMA SPECT/CT (n, %)	Negative Tc-99m-PSMA SPECT/CT (n)
0.2 to <2	18	1 (0.74–1.38)	7 (38.9%)	11
2 to <4	19	3.25 (2.7–3.55)	12 (63.2%)	7
4 to <7	14	5.53 (4.68–6.54)	10 (71.4%)	4
≥7.0	30	13.89 (9.13–22.35)	27 (90%)	3

**Table 3:** Radical prostatectomy group—logistic regression analysis.

**Tc-99m-PSMA SPECT/CT Positivity** **n = 81; PSMA+ = 56**				
**Predictor**	**Univariate ** ** OR (95% CI)**	***p***	**Multivariate ** ** aOR (95% CI) ** ** (Log2(PSA); ISUP Grade)**	***p***
Log2(PSA) (per doubling) *	**1.68** (1.19–2.35)	**0.003**	**1.62** (1.14–2.30)	**0.007**
ISUP grade (per 1-grade increase)	1.67 (0.98–2.82)	0.058	1.55 (0.90–2.65)	0.11
PNI	1.66 (0.64–4.32)	0.29	-	
LVI	1.43 (0.54–3.79)	0.48	-	
PSM	2.10 (0.62–7.09)	0.23	-	
ECE	1.92 (0.57–6.52)	0.29	-	
SVI	1.43 (0.51–4.00),	0.5	-	
**Metastatic disease spread** **n = 56; metastatic = 31, local = 25**				
**Predictor**	**Univariate ** ** OR (95% CI)**	***p***	**Multivariate ** ** aOR (95% CI)** **(Log2(PSA); ISUP grade; SVI)**	***p***
Log2(PSA) (per doubling) *	**1.74** (1.19–2.56)	**0.004**	**1.54** (1.02–2.33)	**0.04**
ISUP grade (per 1-grade increase)	1.55 (0.93–2.59)	0.09	1.15 (0.66–2.01)	0.62
PNI	0.94 (0.34–2.59)	0.91	**-**	
LVI	2.00 (0.70–5.70)	0.19	**-**	
PSM	1.93 (0.65–5.75)	0.24	**-**	
ECE	1.50 (0.49–4.57)	0.48	**-**	
SVI	**4.70** (1.60–13.80)	**0.005**	**3.70** (1.19–11.50)	**0.02**

* PSA “per doubling” was modeled using log2(PSA), so a 1-unit increase corresponds to a 2× increase in PSA. Abbreviations: PNI, perineural invasion; LVI, lymphovascular invasion; PSM, positive surgical margin; ECE, extracapsular extension; SVI, seminal vesicle invasion. Odds ratios with statistically significant *p*-values are bolded.

**Table 4:** Radiation therapy group—Tc-99m-PSMA SPECT/CT detection rates of BCR in different PSA groups.

PSA Group (ng/mL)	Patients (n)	PSA Median (IQR)	Positive Tc-99m-PSMA SPECT/CT (n, %)	Negative Tc-99m-PSMA SPECT/CT (n)
2 to <4	12	2.79 (2.44–3.42)	7 (58.3%)	5
4 to <7	14	5.63 (4.93–6.38)	12 (85.7%)	2
≥7.0	25	11.88 (8.09–21.4)	24 (96%)	1

**Table 5:** Radiation therapy group—logistic regression analysis.

**Tc-99m-PSMA SPECT/CT Positivity** **n = 51. PSMA+ = 43**				
**Predictor**	**Univariate ** ** OR (95% CI)**	***p***	**Multivariate ** ** aOR (95% CI) ** ** (Log2(PSA); ISUP Grade)**	***p***
Log2(PSA) (per doubling) *	**2.87** (1.07–7.71)	**0.036**	3.07 (1.12–8.41)	**0.029**
ISUP grade (per 1-grade increase)	0.71 (0.36–1.40)	0.32	0.60 (0.27–1.34)	0.21
**Metastatic disease spread** **n = 43; metastatic = 16, local = 27**				
**Predictor**	**Univariate ** ** OR (95% CI)**	***p***	**Multivariate ** ** aOR (95% CI)** **(Log2(PSA); ISUP grade)**	***p***
Log2(PSA) (per doubling) *	**2.32** (1.10–4.86)	**0.026**	**2.35** (1.11–4.97)	**0.025**
ISUP grade (per 1-grade increase)	0.93 (0.54–1.59)	0.77	0.88 (0.49–1.58)	0.664

* PSA “per doubling” was modeled using log2(PSA), so a 1-unit increase corresponds to a 2× increase in PSA. Odds ratios with statistically significant *p*-values are bolded.

## Data Availability

While data used for generating results is not publicly available due to ethical restrictions and patient confidentiality, it can be requested if needed from corresponding author.

## Abbreviations

The following abbreviations are used in this manuscript:

BCR	Biochemical recurrence
PSA	Prostate-specific antigen
GS	Gleason score
ISUP	International Society of Urological Pathology
MRI	Magnetic resonance imaging
CT	Computed tomography
PSMA	Prostate-specific membrane antigen
PET/CT	Positron emission tomography with computed tomography
SPECT/CT	Single-photon emission computed tomography
ADT	Androgen-deprivation therapy
PNI	Perineural invasion
LVI	Lymphovascular invasion
PSM	Positive surgical margin
ECE	Extra-capsular extension
SVI	Seminal vesicle invasion
